# Supplementary material for: Effect of Degradation of Polylactic Acid (PLA) on Dynamic Mechanical Response of 3D Printed Lattice Structures
Source: Materials (Basel). 2024 Jul 25;17(15):3674. doi: 10.3390/ma17153674 (PMC11312661; doi:10.3390/ma17153674)
Supplement: Supplementary file 1 [file materials-17-03674-s001.zip › materials-3104619-supplementary.pdf]

Supplementary Materials

S.1. Dimensions of the dog-bone specimens

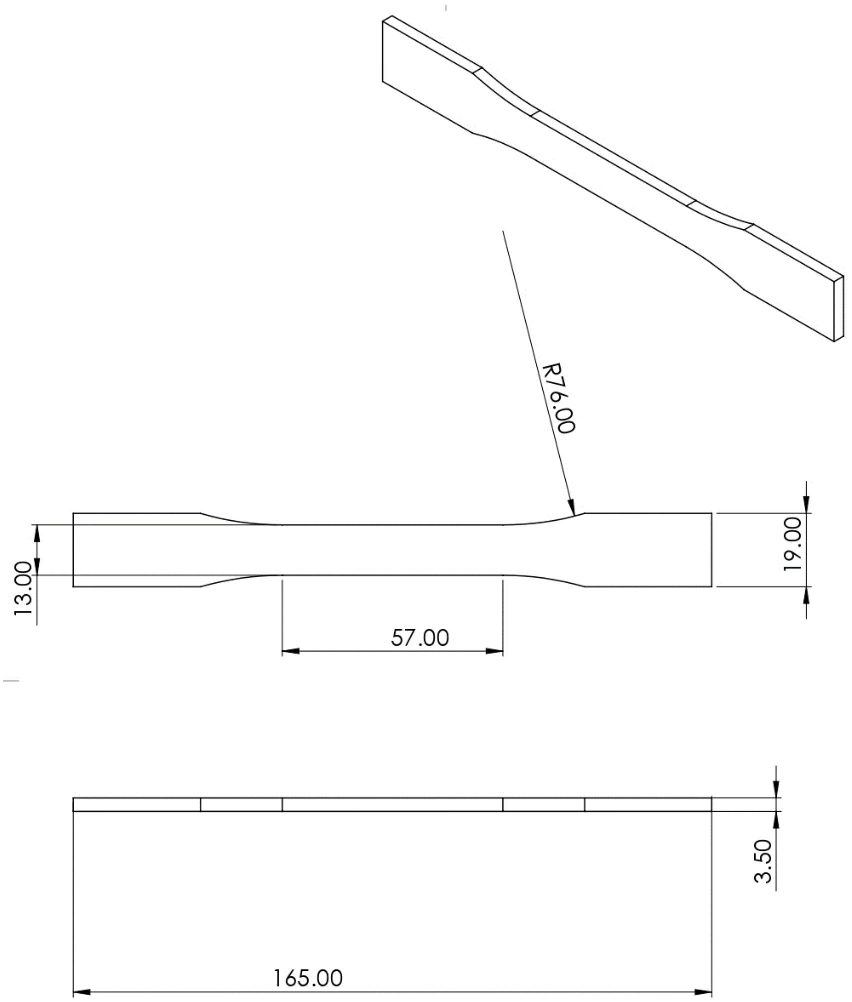

Figure S1: Dimensions of the dog-bone specimen

S.2. Internal energy variations for highest and lowest failure strain levels

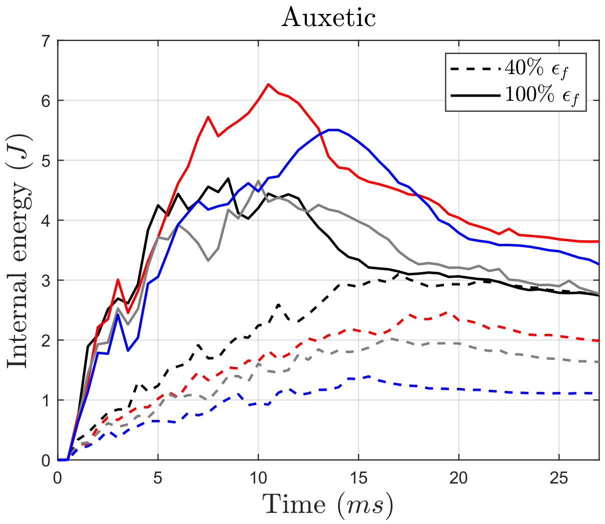

(a)

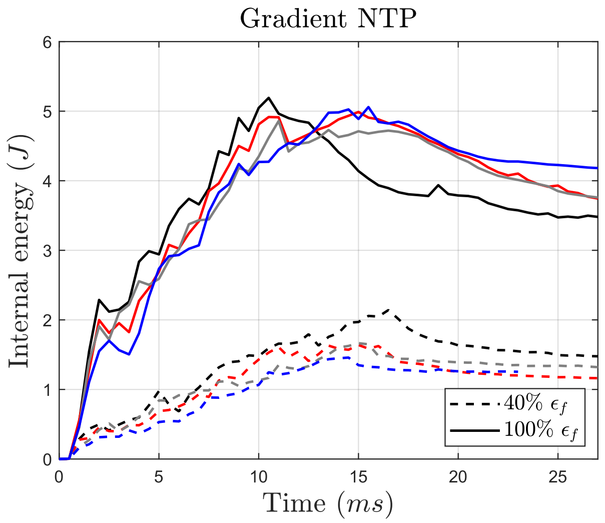

(b)

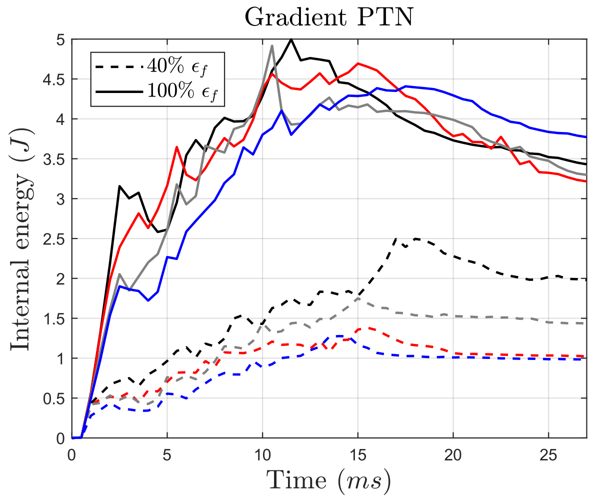

(c)

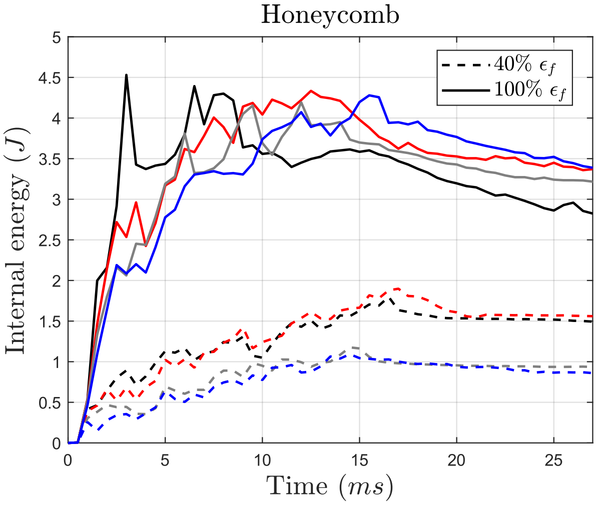

(d)

Figure S2. Variation of the internal energy over time for (a) auxetic, (b) gradient NTP, (c) gradient PTN, and (d) honeycomb core types having different levels of yield stress with the highest and lowest failure strain levels for core material (Black: 100% $\boldsymbol{\sigma_y}$, Red: 80% $\boldsymbol{\sigma_y}$, Gray: 60% $\boldsymbol{\sigma_y}$, Blue: 40% $\boldsymbol{\sigma_y}$).

S.3. Force-displacement curves for highest and lowest failure strain levels

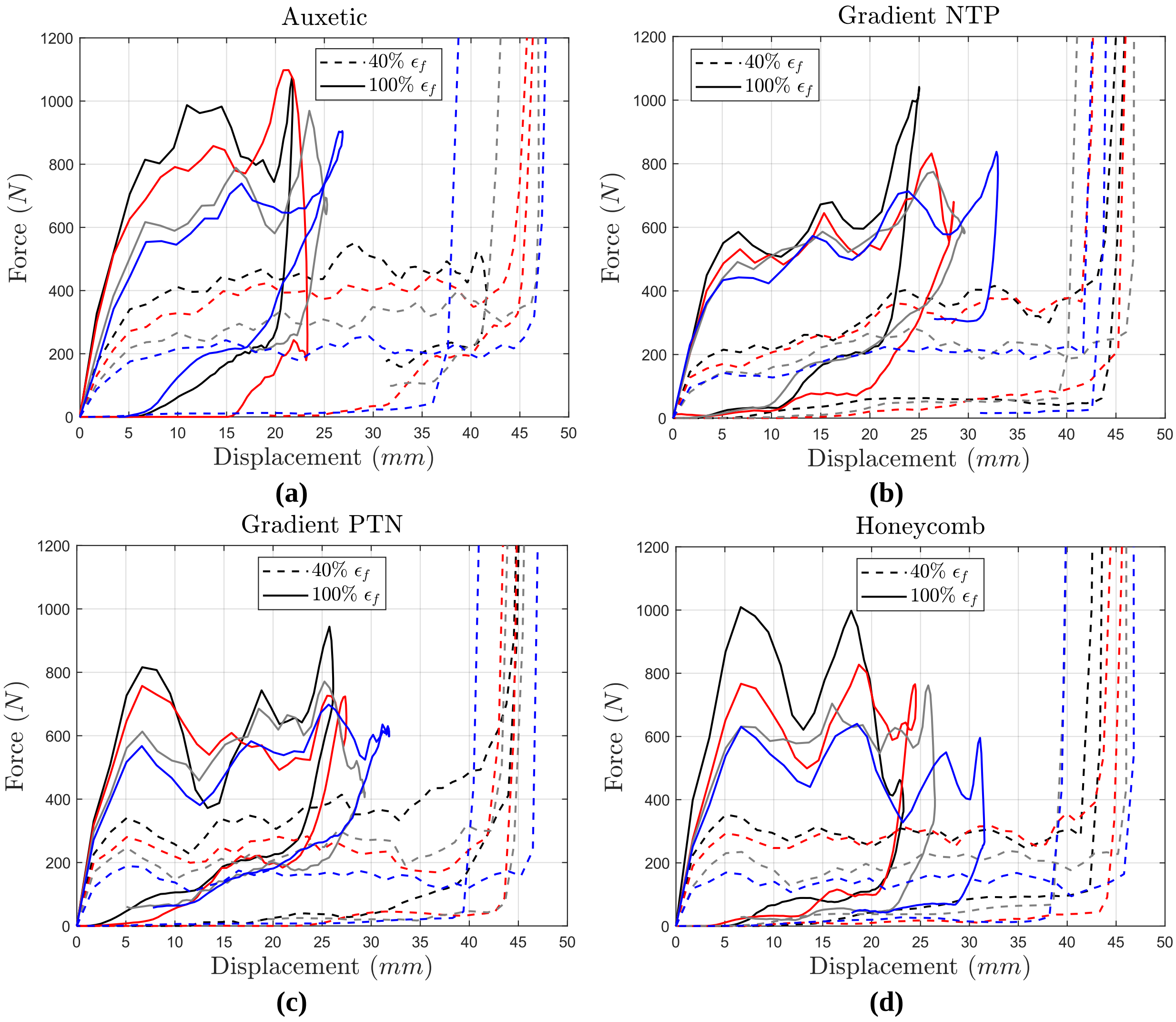

Figure S3. Variation of contact force per impactor's displacement for (a) auxetic, (b) gradient NTP, (c) gradient PTN, and (d) honeycomb core types having different levels of yield stress with the highest and lowest failure strain levels for core material (Black: 100% $\boldsymbol{\sigma_y}$, Red: 80% $\boldsymbol{\sigma_y}$, Gray: 60% $\boldsymbol{\sigma_y}$, Blue: 40% $\boldsymbol{\sigma_y}$).

S.4. von Mises stress distribution for the honeycomb structures

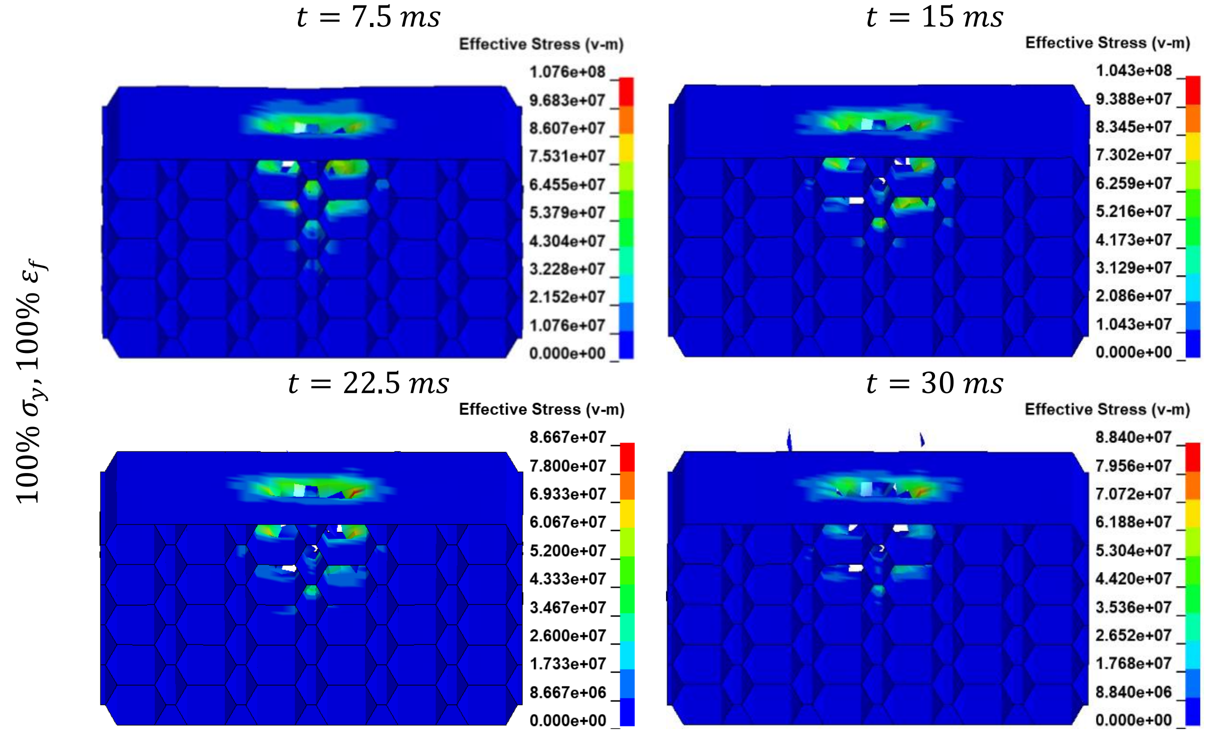

(a)

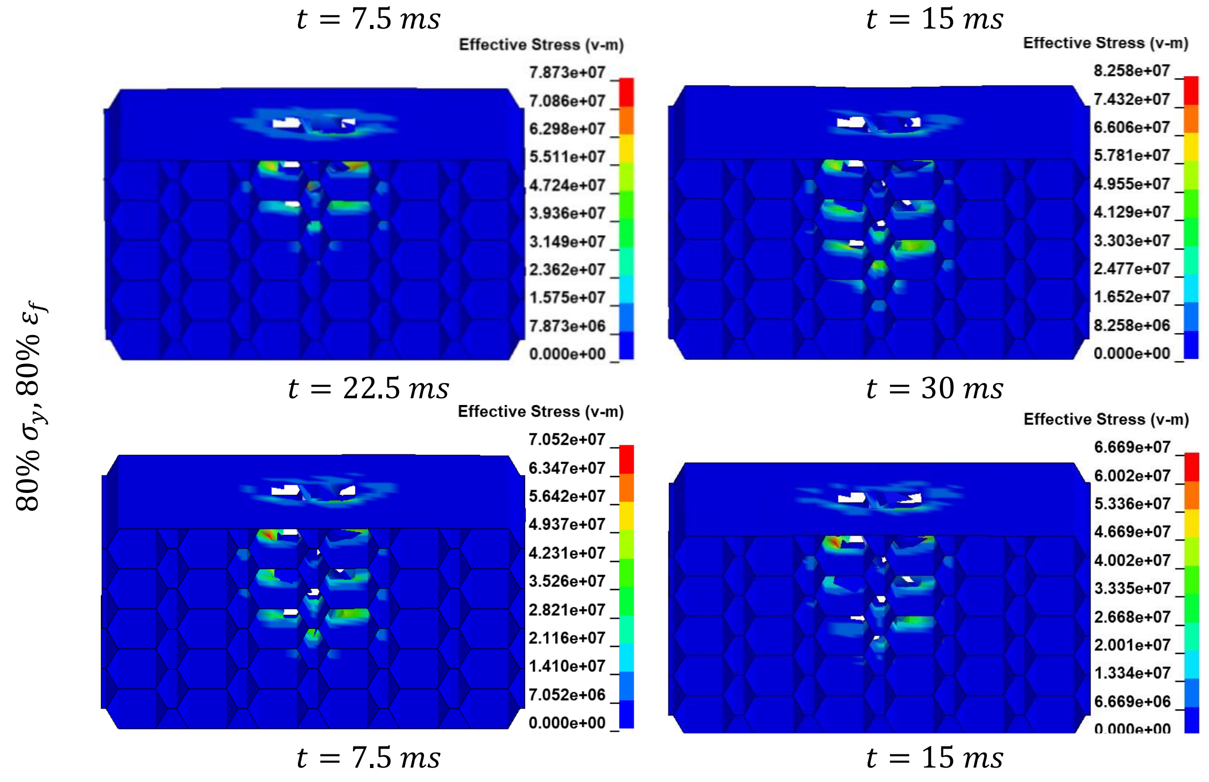

(b)

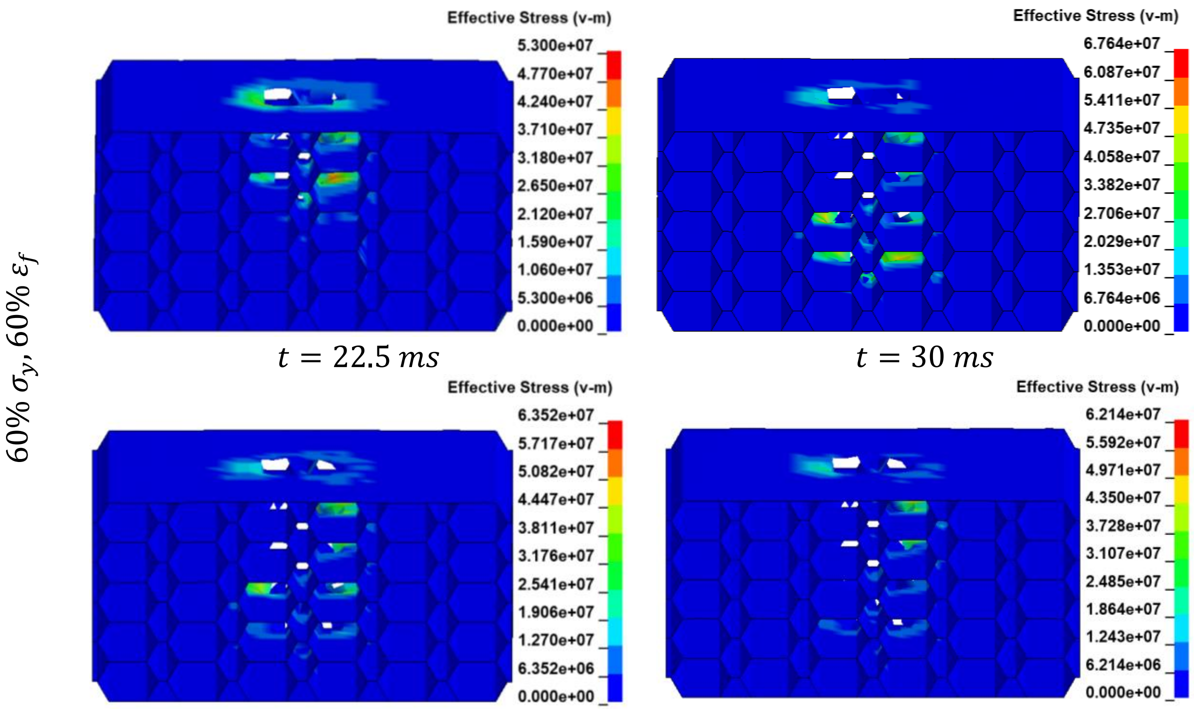

(c)

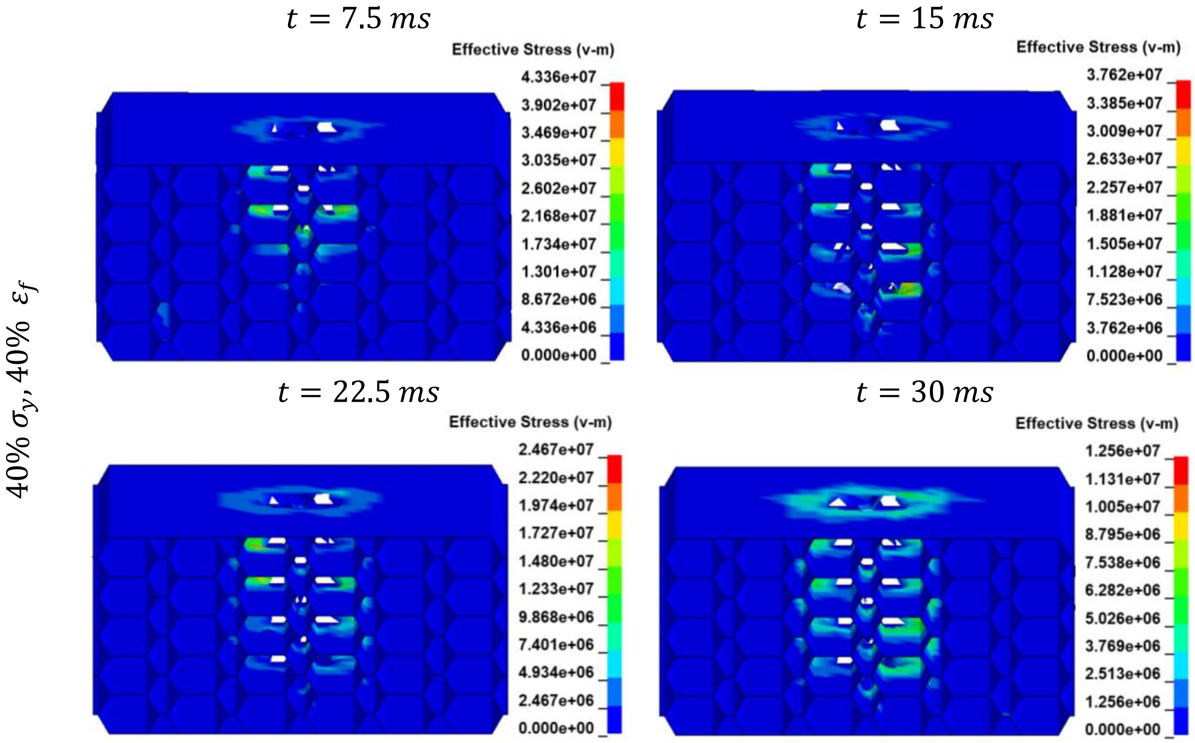

(d)

Figure S4. von Mises stress distribution for the honeycomb structure made up of PLAs having yield strengths and failure strains equal to 40%, 60%, 80%, and 100% of those in the just-printed PLA at $t = 7.5\ ms$, $t = 15\ ms$, $t = 22.5\ ms$, and $t = 30\ ms$.
